# Terahertz Asymmetric S-Shaped Complementary Metasurface Biosensor for Glucose Concentration

**DOI:** 10.3390/bios12080609

**Published:** 2022-08-06

**Authors:** Ibraheem Al-Naib

**Affiliations:** Biomedical Engineering Department, College of Engineering, Imam Abdulrahman Bin Faisal University, Dammam 34212, Saudi Arabia; iaalnaib@iau.edu.sa

**Keywords:** biosensing, terahertz technology, glucose sensing, metasurfaces, bound states in the continuum

## Abstract

In this article, we present a free-standing terahertz metasurface based on asymmetric S-shaped complementary resonators under normal incidence in transmission mode configuration. Each unit cell of the metasurface consists of two arms of mirrored S-shaped slots. We investigate the frequency response at different geometrical asymmetry via modifying the dimensions of one arm of the resonator. This configuration enables the excitation of asymmetric quasi-bound states in the continuum resonance and, hence, features very good field confinement that is very important for biosensing applications. Moreover, the performance of this configuration as a biosensor was examined for glucose concentration levels from 54 mg/dL to 342 mg/dL. This range covers hypoglycemia, normal, and hyperglycemia diabetes mellitus conditions. Two sample coating scenarios were considered, namely the top layer when the sample covers the metasurface and the top and bottom layers when the metasurface is sandwiched between the two layers. This strategy enabled very large resonance frequency redshifts of 236.1 and 286.6 GHz that were observed for the two scenarios for a 342 mg/dL concentration level and a layer thickness of 20 μm. Furthermore, for the second scenario and the same thickness, a wavelength sensitivity of 322,749 nm/RIU was found, which represents a factor of 2.3 enhancement compared to previous studies. The suggested terahertz metasurface biosensor in this paper could be used in the future for identifying hypoglycaemia and hyperglycemia conditions.

## 1. Introduction

One of the most important metabolic disorders is diabetes mellitus. It is considered one of the major causes of death around the world [1,2,3,4]. The two main types of diabetes are caused by the pancreas failing to secrete enough insulin (type 1), or when body cells inappropriately respond to insulin (type 2) [4,5,6]. When fasting, the normal glucose level should be between 70 and 120 mg/dL. However, with glucose levels that are smaller or larger than this range, the patient is identified as having hypoglycemia and hyperglycemia, respectively [6,7,8,9,10]. Failing to maintain glucose levels in the aforementioned range will result in many consequences such as cardiovascular diseases, renal failure, and peripheral neuropathy [3]. Hence, monitoring the glucose level is vital to avoid such complications [11]. Invasive, minimally invasive, and non-invasive devices have been engineered to measure that [3,4,12,13,14,15]. It is important to note that the prevalence of diabetes is predicted to increase to 700 million cases in 20 years [16], which indicates the need to develop new sensors in order to help diabetics. Remarkably, a plethora of different techniques and sensors have been proposed [3,12,17,18]. More specifically, optical sensors have attracted a lot of attention and were designed at different wavelengths [4]. Recently, terahertz (THz) technology has drawn a lot of interest as a potential technique in this regard. It features about 4.14 meV photon energy at 1 THz, and hence it is completely safe for users. Interestingly, within the THz band, various biomolecules have shown clear spectral fingerprints [19,20,21,22]. Therefore, it enables label-free sensing for different analytes and samples [21,23,24,25].

Nevertheless, to realize label-free THz sensors with high sensitivity, a serious challenge has to be addressed. It is represented by the large difference between the THz wavelength and the analyte thickness or quantity. Hence, the electromagnetic field has to be confined in order to increase the THz wave-sample interaction. Several engineered metasurface structures have been suggested to achieve that [26,27,28,29,30,31,32,33]. Conventionally, a redshift of the resonance frequency occurs as a result of dielectric environment modification when the metasurface is coated with an unidentified sample. This shift is utilized as a metric for its refractive index and can be used to identify the analyte type or its concentration. Some sensors have already been proposed to measure the glucose concentration at THz frequencies [34,35,36,37]. In Ref. [34], the authors investigated the optical properties of real samples of blood at terahertz frequencies at different glucose level concentrations. Moreover, in Ref. [35], the authors studied the frequency shift of a simple cross-shaped metasurface on PET substrate when it is immersed in the blood samples. This configuration showed the potential of THz metasurfaces for glucose sensing. Furthermore, the authors in Ref. [36] utilized asymmetric split-ring resonators of thick metals on a silicon dioxide substrate, but the achieved sensitivity is quite low. Generally speaking, various techniques have been suggested to achieve as large a light–matter interaction as possible including bound states in the continuum (BIC) [28,33,38,39], Fano resonance [40,41], electromagnetic-induced transparency resonance [42,43,44,45], and some other configurations [30,33,46,47,48,49,50,51,52]. Among these, Fano resonance, which is considered a quasi-BIC resonance, has been of particular interest for many applications, such as lasing spasers [53,54], slow light devices [55,56,57], and sensors [28,30,48,49,58,59,60,61]. Hence, the authors in Ref. [37] proposed a metasurface consisting of a symmetric complementary split rectangular resonator under oblique THz wave excitation. The achieved resonance frequency shift of resonance frequency at 0.506 THz was 122 GHz for a glucose concentration of 342 mg/dL for a 5 μm thick sample, which is quite good as it can be distinguished in the measurements. Nevertheless, the question remains open: How can the performance of such sensors be improved? It is worth mentioning that using free-standing substrate-free metasurfaces of complementary structures using only metal layers has shown very good performance [47,62,63].

Therefore, in this paper, a terahertz metasurface consisting of an array of asymmetric S-shaped complementary resonators is proposed. It is based on the utilization of a quasibound state in the continuum with broken in-plane symmetry. This metasurface can be easily fabricated using a simple optical setup with a laser beam. The unit cell of the metasurface consists of two arms of mirrored S-shaped slots. The transmission and reflection frequency response of this configuration was studied with different geometrical asymmetry. Moreover, the electric field spatial distributions have been examined. More notably, to cover a wide range of glucose levels from hypoglycemia to hypercalcemia conditions, the response to different glucose concentrations when the top layer or top and bottom layers are discussed for different sample thicknesses. Moreover, we examine the wavelength sensitivity of the suggested metasurface sensor by simulating different settings with a range of the refractive index of the sample layers.

## 2. Sensor Configuration and Simulation Methodology

**Figure 1:**
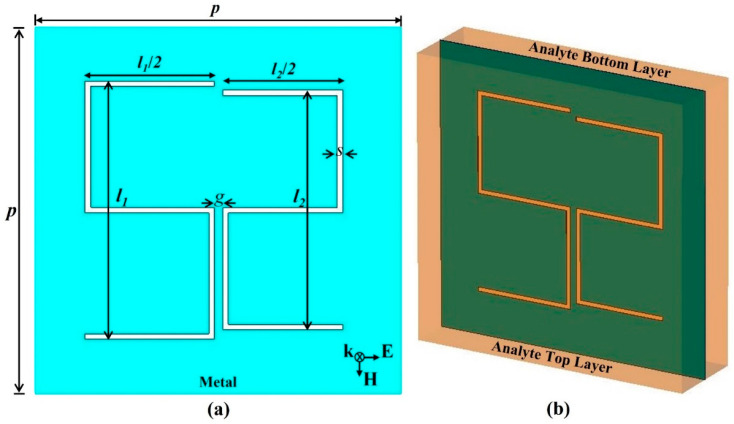
(**a**) Unit cell dimensions of the asymmetric S-shaped complementary resonator with the related dimensions. (**b**) Three-dimensional representation showing the glucose sample top and bottom layers. The inset of part (**a**) illustrates the field polarization.

**Figure 2:**
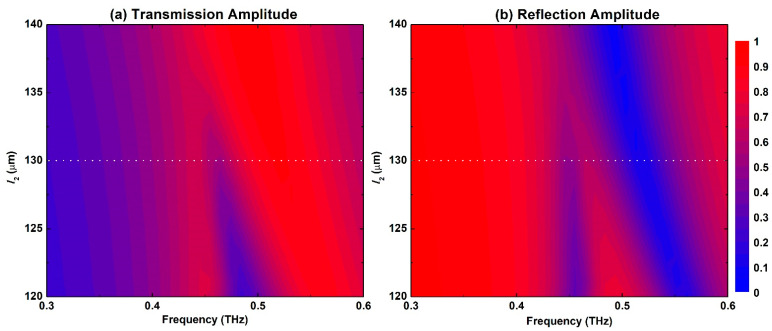
(**a**) Transmission and (**b**) reflection spectral map response of the S-shaped metasurface for *l_2_* values that swept between 120 μm and 140 μm with 1 μm step.

**Figure 3:**
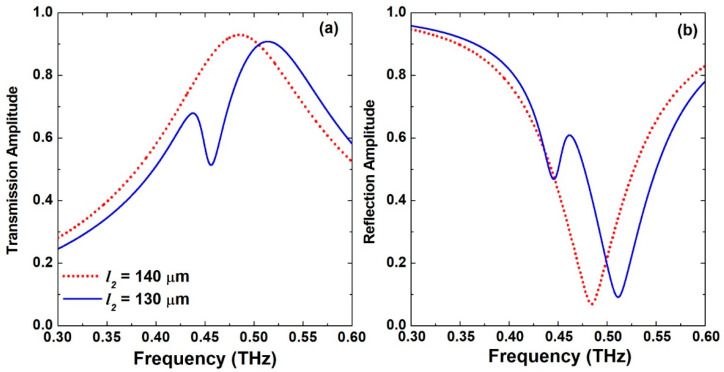
(**a**) Transmission and (**b**) reflection frequency response of the S-shaped complementary metasurface for the symmetric case when *l_2_* = 140 μm and the asymmetric case when *l_2_* = 130 μm.

Figure 1a,b show the Figure 2D and Figure 3D configurations of the unit cell. Each unit cell consists of an S-shaped complementary resonator (SCR) with two arms, one of which is a smaller mirrored version of the other. Figure 1a illustrates the geometrical dimensions of the SCR with the relevant dimensions: the height of the left-hand S-shaped arm of *l_1_* = 140 μm, the height of the right-hand S-shaped arm of *l_2_*, the slot of *s* = 3 μm, the gap between the two arms of *g* = 4.5 μm, and periodicity of *p* = 200 μm. Moreover, the aluminum metallic layer thickness is taken to be 200 nm. Furthermore, it is worth noting that the adopted dielectric constant values have been taken from Ref. [35], which were taken originally from Ref. [34], by characterizing real blood samples. The values cover different frequencies of a wide range of glucose level concentrations.

Figure 1b depicts the glucose top and bottom layers as the metasurface can be coated with the glucose sample or immersed in the middle of it. The simulation package Computer Simulation Technology (CST) Microwave Studio [64] was utilized to carry out all the simulations. It solves Maxwell equations in the frequency domain based on a finite integration technique. Excitation of a plane wave was considered with periodic boundary conditions to mimic the actual scenario. The inset of Figure 1a shows the field polarization where the electric component is horizontally oriented. Hence, the excitation of a symmetric dipole resonance mode is expected as the THz waves are exciting the metasurface under normal incidence.

## 3. Results and Discussion

Figure 2 displays the maps of the transmission and reflection amplitude spectral response for the proposed metasurface for a sweep of the *S*-shape right-hand arm *l_2_* dimension between 120 μm and 140 μm with a step of 1 μm. When *l_2_* = *l_1_* = 140 μm, the structure is completely symmetric and hence only the dipole resonance is excited. It is featured by a broad response with a peak in the transmission amplitude and a dip in the reflection amplitude as shown in Figure 2a,b, respectively. The spectral response of this symmetric configuration is also presented by the dotted red lines in Figure 3. The dipole resonance frequency dip in the reflection amplitude is excited at 0.484 THz and is symmetric with a very broad spectral response. This case also corresponds to the symmetry-protected BIC situation. Once the dimension *l_2_* is decreased, the structure becomes asymmetric, and a small amplitude asymmetric resonance is excited that becomes visible in Figure 2 when *l_2_*= 135 μm. The symmetry breaking allows the radiation of an asymmetric resonance mode into the far field. This asymmetric mode is also called Fano-like resonance, which is a special case of BIC called quasi-BIC [38]. Moreover, the peak in the transmission amplitude is blue-shifted gradually as well as the dip in the reflection amplitude as a result of modifying the electrical length of the two arms of the resonator. Further decreasing *l_2_* leads to broadening of the asymmetric resonance and its spectral bandwidth. Hence, its quality (*Q*-) factor, which is defined as the ratio of resonance frequency to the bandwidth, is decreased. A small *Q*-factor is correlated with low field confinement and, hence, less field–sample interaction. In order to investigate the performance of this metasurface for biosensing, a value of *l_2_* = 130 μm was selected as indicated by the dotted white lines shown in Figure 2a,b. The exact transmission and reflection spectral response for this configuration is shown in Figure 3 as solid blue lines with very clear asymmetric resonance. The asymmetric resonance dip in the reflection response is excited at 0.446 THz as shown in Figure 3b. This configuration was adopted for all the analyses discussed below. It represents a trade-off between the amplitude of the resonance and its sharpness in order to facilitate reliable measurements for a practical dynamic range of the THz spectrometers that are available and the measurable scan time window.

**Figure 4:**
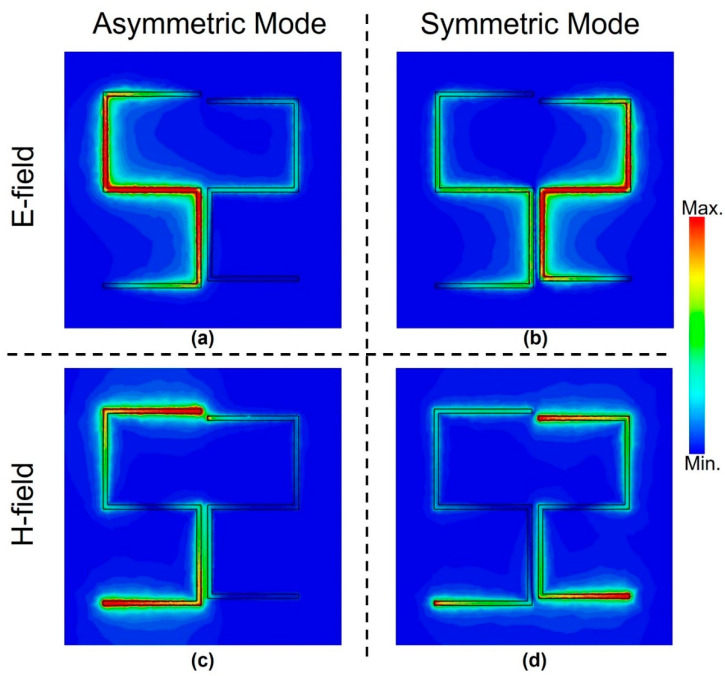
Spatial distributions of electric fields (**a**) and (**b**)**,** magnetic fields (**c**) and (**d**) at the surface of the metasurface for the asymmetric resonance (**a**) and (**c**), and symmetric dipole resonance (**b**) and (**d**) modes.

To get an understanding of the resonance excitation, additional simulations to visualize the electromagnetic fields were carried out for both symmetric and asymmetric resonance frequencies as shown in Figure 4 when *l_2_* = 130 μm. It presents the spatial distributions of electric (E) and magnetic (H) fields at the surface of the structure for the Fano-like asymmetric resonance and symmetric dipole resonance modes. It is evident that the electric and magnetic fields complement each other when it comes to the excitation location. For instance, the E-field displayed in Figure 4a is confined and maximum in the vertical slots as well as the horizontal middle slot of the left arm for the asymmetric mode. However, the H-field is shown in Figure 4c, and it is confined and maximum in the upper and lower horizontal slots of the left arm. Similar observations can be made for the symmetric mode shown in Figure 4b,d for the electric and magnetic fields, respectively. However, it is evident that both arms are excited in the latter case, and the excitation is higher in the right arm compared to the left one as this arm represents a smaller electrical length and is hence related to the higher frequency of the dipole resonance mode compared to the asymmetric resonance mode. Visualizing the fields not only helps in understanding the behavior of the resonator but also helps in using less analyte to coat some locations only where the field is highly confined. This way, the THz wave–sample interaction is optimized, and hence the achieved sensitivity can be as good as covering the whole structure, but with much less sample material [25].

**Figure 5:**
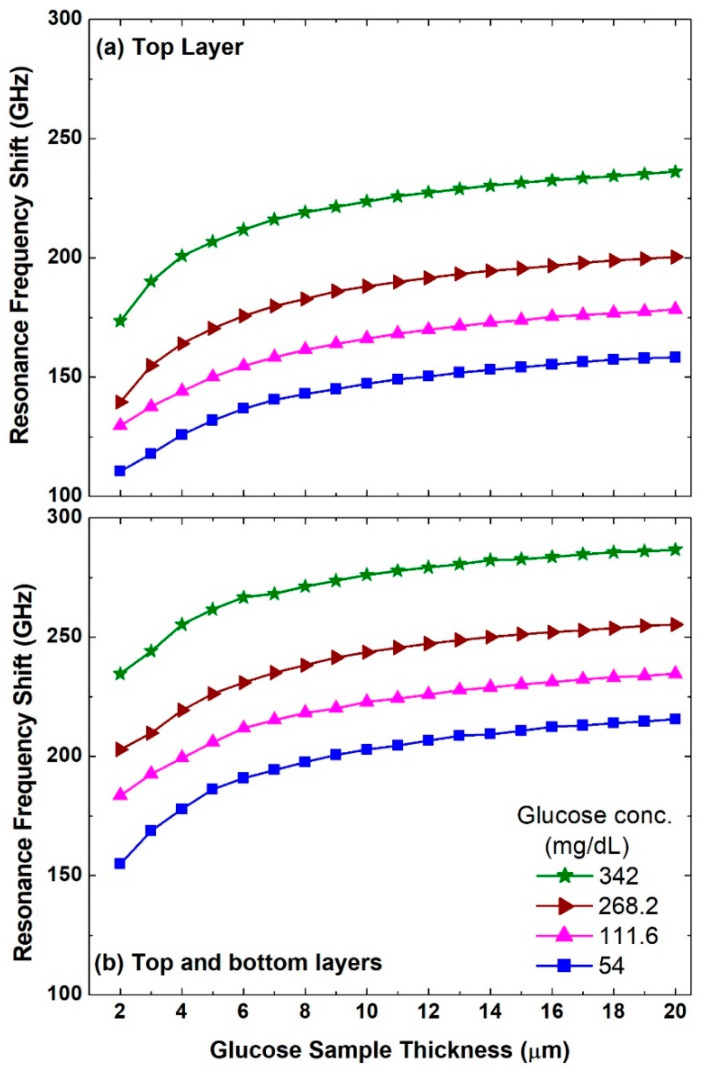
Asymmetric resonance frequency shifts when (**a**) the top layer is considered and when (**b**) the top and bottom layers are considered for different glucose concentration levels and a sweep of glucose sample thicknesses between 2 and 20 μm.

Next, we examine the performance of this metasurface as a biosensor for different glucose samples with various glucose concentration levels covering hypoglycemia, normal, and hyperglycemia conditions from 54 to 342 mg/dL. As the sample with each concentration level has a unique dielectric constant value, the dielectric environment of the metasurface will be modified and this leads to a redshift in the resonance frequency. Two scenarios have been considered, namely (i) when the glucose sample is applied as a top layer of the metasurface and (ii) when the metasurface is immersed in the glucose sample, i.e., sandwiched between the top and bottom layers of the glucose sample as shown in Figure 1b. In both scenarios, the sample thickness of each layer was swept from 2 to 20 μm. Therefore, it is expected that there will be a minimum and maximum redshift in the resonance frequency when sample thickness = 2 μm for a glucose concentration level of 54 mg/dL and sample thickness of 20 μm for the concentration level of 342 mg/dL, respectively. The results of the two scenarios are presented in Figure 5a,b, respectively. It is observed that even with a sample thickness of 2 μm and a concentration of 54 mg/dL, there is a significant redshift of 110.6 GHz as shown in Figure 5a. Moreover, the corresponding redshift with 20 μm sample thickness and a concentration of 342 mg/dL is 236.1 GHz. This shows the potential of this sensor to measure a small amount of glucose sample with small concentration levels in the hypoglycemia range. The latter is deemed to be a serious condition for diabetic people [65]. Increasing the thickness of the sample and hence the amount of the sample as presented in Figure 5a leads to an increase in the redshift of the asymmetric resonance mode. More importantly, different concentration levels such as 111.6, 268.2, and 342 mg/dL show a distinct frequency shift and hence can be discerned from each other quite easily.

It is also noted that the redshift value starts to saturate beyond a thickness of 10 μm. Figure 5b for the top and bottom layers scenario reveals a clear improvement in the performance as the resonance frequency shift is larger compared with the top layer scenario shown in Figure 5a. The redshift with 20 μm sample thickness and a concentration of 342 mg/dL reaches 286.6 GHz. These results reveal an important advantage of this free-standing sensor as it can be immersed in the sample. To implement such a scenario experimentally, a possible configuration would require a cuvette with a thickness in the propagation direction equal to the top and bottom layers’ thickness. Hence, the free-standing metasurface can be placed in the middle of such a cuvette.

**Figure 6:**
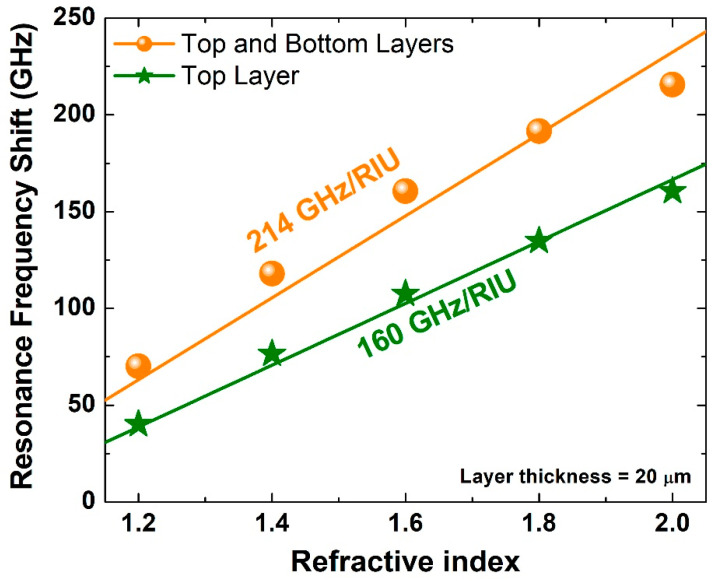
Asymmetric resonance frequency shift for two scenarios (**i**) top layer and (**ii**) top and bottom layers for different values of the refractive index between 1.2 and 2.0 with each sample layer thickness being 20 μm.

Next, a traditional assessment of sensing capability was performed. A sample of the maximum thickness of 20 μm that was used in the previous analysis was considered with a range of refractive index between 1.2 and 2.0. Figure 6 shows asymmetric resonance frequency shift for the two scenarios: (i) top layer and (ii) top and bottom layers scenarios. One of the metrics of design sensitivity that is routinely reported is the slope of the fitting lines of such results, i.e., the resonance frequency shift in GHz per refractive index unit (RIU). As this slope increases, it becomes easier to differentiate between two analytes with close glucose concentration levels. Hence, it is always desirable to increase this slope. The top layer scenario results are presented in Figure 6 by stars and a green fitting line with a slope of 160 GHz/RIU, and the top and bottom layers scenario results are shown as circles and an orange fitting line with a slope of 214 GHz/RIU. Since the latter number is much larger than the former one, the top and bottom layer scenario has a clear advantage over the top layer scenario.

However, these numbers can increase or decrease if the exact resonance frequency is larger or smaller than the one of the current design, respectively. A more comprehensive approach is to use the wavelength sensitivity, which is conventionally calculated using the following formula [25,48]:

(1) $$S=\left|\frac{d\lambda \;}{dn}\right|=\frac{\Delta f}{\Delta n}\times \frac{c_{o}}{f_{r}^{2}}$$

where Δf is the asymmetric resonance frequency shift, Δn is the difference in the refractive index, $c_{o}$ is the speed of light in free space, and $f_{r}$ is the resonance frequency. Calculating the sensitivity using this equation offers a fair assessment as the resonance frequency is incorporated in the evaluation. The analysis revealed a very high sensitivity level of 241,308 and 322,749 nm/RIU for the design proposed in this paper for both scenarios, respectively. Simulating the proposed design in Ref. [37] with an overlayer thickness of 20 μm revealed a wavelength sensitivity of 140,000 nm/RIU. Hence, a factor of 2.3 enhancement was achieved using the proposed design in this paper compared with the achieved wavelength sensitivity in the previous study. It is worth mentioning that for a fair comparison among different configurations and sensors, one should consider at least ten different parameters. A detailed discussion about these parameters and their effect can be found in Ref. [37]. Nevertheless, the novelty of the proposed design in this paper is based on three aspects. It is (i) freestanding and hence it can be easily fabricated. Moreover, (ii) it works under normal incidence, and this enables simple measurement configuration and procedures. Furthermore, (iii) it offers very high wavelength sensitivity. Hence, excellent performance can be achieved with a simple fabrication process and conventional experimental setup based on the transmission mode configuration. It is also worth mentioning that the quasi-BIC in this study is in the geometry space, which is rather different compared to the design in the previous study [37] that was in the momentum space.

## 4. Conclusions

In conclusion, a terahertz metasurface biosensor was proposed based on an asymmetric S-shaped complementary resonator for glucose concentration level sensing. Moreover, the design configuration is a free-standing metasurface, and hence a layer of the sample can be applied as a top layer above the metasurface, or the metasurface can be immersed inside the sample with a layer on the top and another one on the bottom. When the top layer of the sample as small as 2 μm is considered with the glucose concentration level of 54 mg/dL applied as an overlayer on the top, the resulting redshift in the resonance frequency is 110.6 GHz, which is a significant shift that can be easily identified. Moreover, increasing the glucose concentration level to 342 mg/dL and using 20 μm thick layers, one on the top and another one on the bottom, increased the redshift to 286.6 GHz, which is almost three times compared with a 2 μm sample and concentration of 54 mg/dL. Furthermore, a very high wavelength sensitivity was accomplished of more than 322,000 nm/RIU. Therefore, this design may pave the way to building glucose concentration level biosensors that can be easily fabricated and have high performance.

## Data Availability

Not applicable.
